# Early onset Arboleda-Tham syndrome due to *KAT6A* variants: Case report

**DOI:** 10.3389/fgene.2025.1704725

**Published:** 2025-12-08

**Authors:** Hongbo Chen, Dan Yu, Shanling Liu, Hanbing Xie, Lina Qiao, Hanmin Liu, Weiran Li

**Affiliations:** 1 Department of Medical Genetics/Prenatal Diagnostic Center, West China Second University Hospital, Sichuan University, Chengdu, China; 2 Key Laboratory of Birth Defects and Related Diseases of Women and Children (Sichuan University), Ministry of Education, Chengdu, Sichuan, China; 3 Department of Pediatrics, West China Second University Hospital, Sichuan University, Chengdu, China; 4 Department of Pediatric Pulmonology and Immunology, West China Second University Hospital, Sichuan University, Chengdu, China; 5 NHC Key Laboratory of Chronobiology (Sichuan University), Chengdu, China; 6 Department of Pediatric Pulmonology and Immunology, WCSUH-Tianfu Sichuan Provincial Children’s Hospital, Sichuan University, Meishan, China; 7 The Joint Laboratory for Lung Development and Related Diseases of West China Second University Hospital, Sichuan University and School of Life Sciences of Fudan University, West China Institute of Women and Children’s Health, West China Second University Hospital, Sichuan University, Chengdu, China; 8 Sichuan Birth Defects Clinical Research Center, West China Second University Hospital, Sichuan University, Chengdu, China

**Keywords:** KAT6A, Arboleda-Tham syndrome, early onset, arrhythmias, seizures

## Abstract

**Background:**

Arboleda-Tham syndrome (ARTHS), caused by likely pathogenic or pathogenic variants in the *KAT6A* gene, is characterized by developmental delay, distinctive facial dysmorphic features, and congenital cardiac anomalies. ARTHS warrants consideration in the differential diagnosis of neonates exhibiting unexplained cardiac arrhythmias, seizures, and dysmorphic features, although neonatal-onset manifestations remain underrecognized.

**Case:**

We report two Chinese patients with *KAT6A* variants diagnosed in the neonatal period who presented with life-threatening manifestations. Two unrelated neonates presented with severe cardiac arrhythmias or seizures within the first month of life, in association with congenital heart defects and developmental delay. Whole-exome sequencing (WES) identified two *de novo KAT6A* variants: a novel splice-site variant (c.3352 + 1G>C) in patient 1, who developed supraventricular tachycardia at 23 days of life, and a previously reported missense variant (c.4645G>A; p. Gly1549Ser) in patient 2 with seizures onset at 11 days. Both patients exhibited complex congenital heart disease (Patient 1: VSD, ASD and PDA; Patient 2: PFO and PDA), developmental delay, and characteristic dysmorphic features consistent with ARTHS.

**Conclusion:**

This report highlights the critical role of genomic sequencing in the diagnostic evaluation of neonates with unexplained cardiac arrhythmias or seizures. WES should be considered in neonates exhibiting severe early-onset multisystem involvement and dysmorphic features to investigate potential *KAT6A* variants. These findings substantially expand the phenotypic spectrum of ARTHS by documenting severe neonatal manifestations and contribute to a deeper understanding of KAT6A-related phenotypic variability.

## 1 Introduction

Arboleda-Tham syndrome (ARTHS; OMIM: 616268), alternatively termed KAT6A syndrome, is recognized as a rare autosomal dominant neurodevelopmental disorder caused by variants in the *KAT6A* gene (OMIM: 601408) located on chromosome 8p11.21 (Arboleda et al., 2015). The *KAT6A* gene encodes a histone acetyltransferase of the MYST family, which is essential for chromatin remodeling, transcriptional regulation, and embryonic development (Avvakumov and Côté, 2007). Since its initial characterization by Arboleda et al. in 2015, more than 150 cases have been documented worldwide, significantly broadening the known phenotypic spectrum of this disorder (Kennedy et al., 2019). KAT6A syndrome demonstrates marked clinical heterogeneity, with characteristic manifestations including intellectual disability, global developmental delay, hypotonia, distinctive facial dysmorphism, and multisystem involvement (Tham et al., 2015). Neurological involvement is particularly significant, with seizure disorders documented in 20%–30% of cases, although the age of onset and clinical severity demonstrate substantial variability (Millan et al., 2016). Additional clinical features may include feeding difficulties, gastrointestinal dysmotility, ophthalmologic anomalies, and increased susceptibility to recurrent infections (Zwaveling-Soonawala et al., 2017).

While the majority of KAT6A syndrome cases manifest during infancy or early childhood with developmental delays, neonatal presentations remain less comprehensively characterized in the literature. Cardiac arrhythmias during the neonatal period have been rarely reported in association with *KAT6A* variants, though structural cardiac anomalies are well-documented features of the syndrome (Huang et al., 2016). Similarly, while seizure disorders are recognized components of the phenotypic spectrum, neonatal-onset seizures have been infrequently documented and require more systematic documentation and further investigation (Wiesel-Motiuk and Assaraf, 2020).

This study describes two neonates with likely pathogenic or pathogenic *KAT6A* variants exhibiting severe phenotypic manifestations during the neonatal period. These cases contribute to the growing recognition of the potential for early neonatal presentation of ARTHS, although the direct causative relationship between neonatal arrhythmias and/or seizures and *KAT6A* variants remains to be validated through larger cohort studies. They underscore the clinical imperative to include this diagnosis in the differential evaluation of neonates presenting with severe cardiorespiratory instability or refractory neurological symptoms, thereby enabling timely molecular confirmation and the implementation of targeted management approaches.

## 2 Case description

### 2.1 Case 1

The proband, a female infant, was delivered via vaginal birth at 35 weeks gestation with anthropometric measurements within normal ranges (Table 1). Neonatal course was complicated by feeding difficulties and apnea, necessitating a 10-day admission to the neonatal intensive care unit (NICU). At 23 days of life, the patient was transferred to our institution’s NICU following episodes of paroxysmal supraventricular tachycardia (SVT), which were successfully managed with adenosine. Subsequent 24-h Holter monitoring revealed ventricular premature contractions and evidence of a Wolff-Parkinson-White pattern, with recurrent arrhythmic events documented during the first postnatal months. Echocardiographic evaluation identified multiple congenital cardiac anomalies including perimembranous ventricular septal defect (VSD), secundum atrial septal defect (ASD), and patent ductus arteriosus (PDA), accompanied by pulmonary hypertension. Definitive surgical correction (combined procedure consisting of surgical repair of Ventricular Septal Defect, Mitral Valve Repair, and Resection of Subaortic Membrane) was performed at 9 months of age. During subsequent follow-up through 5 years of age, occasional premature beats were detected, but no recurrent episodes of sustained supraventricular tachycardia requiring intervention occurred.

**TABLE 1:** Clinical characteristics of the patients.

Items	Case 1	Case 2
National	China	China
Gender	Female	Male
Birth characteristics		
Mode of delivery	Vaginal delivery	Cesarean section
Week of gestation	35	36^+4^
Birth weight (g)	2,580 (p75-p90)	2,450 (p10-p25)
Birth height (cm)	47 (p50-p75)	48 (p50-p75)
Clinical findings		
Age at the onset	23 days	10 days
Facial abnormalities	Bilateral microphthalmia, ocular hypertelorism, shortened/flattened philtrum	Mild left-sided micrognathia, prominent nasal bridge, high-arched narrow palate
Developmental delay	Rolling over at 7 months Independent standing at 24 months Unassisted ambulation at 36 months Persistent gait instability precluded running at 5 years of age	Lifting his head slightly at 5 months Rolling over at 7 months Independent standing remained absent by 18 months; microcephaly (head circumference:31 cm at birth; 32 cm at 2 months; 37 cm at 6 months) Oro-motor dyspraxia (poor sucking, swallowing, feeding difficulties, orogastric tube feeding)
Intellectual disability	With the first word emerging at 21 months No functional verbal communication established by 5 years	Absent speech, no single words at 12 months
Cardiac system	Paroxysmal supraventricular tachycardia; ventricular premature beats and preexcitation syndrome Perimembranous ventricular septal defect, atrial septal defect, patent ductus arteriosus and pulmonary hypertension	Prenatal period: Narrow of the aortic isthmus; atrial premature beats Neonatal period: Patent foramen ovale and patent ductus arteriosus; atrial premature beats
Nervous system	Cranial MRI at 2 years of age was unremarkable	Prenatal ultrasound: Bilateral choroid plexus cysts in fetus (right: 0.36 cm × 0.24 cm; left: 0.43 cm × 0.28 cm) Electroencephalography in neonatal period: (1) mildly delayed maturation of background activity; (2) several multifocal sharp wave discharges during sleep, predominantly over the right fronto-temporal midline regions Cranial MRI: Mild widening of the extracerebral spaces in the bilateral frontal, temporal, and parietal regions
Respiratory system	Recurrent respiratory tract infections	Neonatal respiratory distress during neonatal period Recurrent respiratory tract infections

The patient exhibited significant global developmental delay, achieving motor milestones substantially later than peers: rolling over at 7 months, independent standing at 24 months, and unassisted ambulation at 36 months. Persistent gait instability precluded running at 5 years of age. Language development was markedly impaired, with the first words emerging at 21 months and no functional verbal communication established by 5 years. Characteristic dysmorphic features included bilateral microphthalmia, ocular hypertelorism, and a shortened/flattened philtrum (Table 1). Formal neurodevelopmental assessment using the Griffiths Mental Development Scale-Chinese (Tso et al., 2018) 2-8 scale at 5 years and 4 months of age revealed significant delays across all domains: Locomotor (A): quotient: 64; Personal-Social (B): quotient: 58; Hearing and Language (C): quotient: 56; Eye and Hand Coordination (D): quotient: 64; Performance (E): quotient: 52; Practical Reasoning (F): quotient: 64. All subscale scores fell significantly below the normative threshold (<−2 SDs, 70).

### 2.2 Case 2

The second proband, a male infant, was born to healthy, non-consanguineous Chinese parents. A prenatal ultrasound at 33 weeks of gestation revealed bilateral choroid plexus cysts along with aortic isthmus narrowing. Subsequent fetal monitoring at 35 weeks detected atrial premature beats. Due to recurrent non-reassuring non-stress test (NST) findings, delivery was accomplished via cesarean section at 36+4 weeks of gestation and the anthropometric parameters are summarized in Table 1.

Immediate NICU admission was required for neonatal respiratory distress syndrome necessitating invasive mechanical ventilation. During the first postnatal year, the patient experienced multiple convulsive episodes.

**FIGURE 1:**
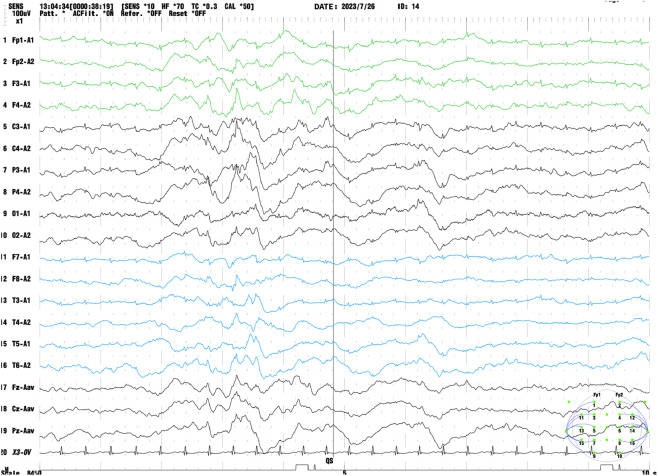
Electroencephalography of case 2. Mildly delayed maturation of background activity and several multifocal sharp wave discharges during sleep, predominantly over the right fronto-temporal midline regions.

Electroencephalography (EEG) demonstrated abnormal neonatal findings, as shown in Figure 1. Cranial magnetic resonance imaging (MRI) revealed mild bilateral frontal, temporal, and parietal extracerebral space widening. Metabolic workup via tandem mass spectrometry showed elevated urinary 4-hydroxyphenyllactate levels, likely reflecting hepatic immaturity. Comprehensive metabolic and endocrine evaluations performed during seizure episodes revealed no evidence of metabolic instability or endocrine dysfunction. Anticonvulsant therapy with levetiracetam was initiated, although seizure control remained suboptimal throughout the course of treatment.

Cardiac evaluation during the neonatal period revealed both a patent foramen ovale (PFO) and PDA. Twenty-four-hour Holter monitoring demonstrated isolated atrial premature beats occurring once per 24-h period. The first year of life was complicated by recurrent respiratory tract infections, including two episodes of pneumonia requiring hospitalization. Distinctive dysmorphic features became apparent by 1 month of age, including mild left-sided micrognathia, a prominent nasal bridge, and high-arched narrow palate.

The patient exhibited significant global developmental delay and intellectual disability. Motor milestones were markedly delayed: lifting his head slightly at 5 months, rolling attained at 7 months and persistent inability to stand independently at 18 months. Additionally, oro-motor dysfunction led to chronic feeding difficulties, necessitating nasogastric tube feeding. Language development was severely impaired, with absence of single words by 12 months of age (Table 1).

### 2.3 Genetic results

This study was conducted with informed consent from all participants’ parents and received ethical approval from the ethics committee of West China Second Hospital of Sichuan University (approval number 2024-269). Genomic DNA was extracted from peripheral blood samples obtained from both probands and their parents for trio-based whole-exome sequencing (trio-WES). Paired-end sequencing was conducted on the Illumina NovaSeq6000 platform, generating 150-bp reads. The reads were aligned to the GRCh38/hg38 reference genome using Burrows-Wheeler Aligner software. To filter out low-frequency variants, we utilized the gnomAD v2.1.1 and the 1000 Genomes Project database. The classification of variants followed the American College of Medical Genetics and Genomics (ACMG) guidelines (Richards et al., 2015). We next focused on identifying pathogenic variants in the *KAT6A* gene in both patients.

**FIGURE 2:**
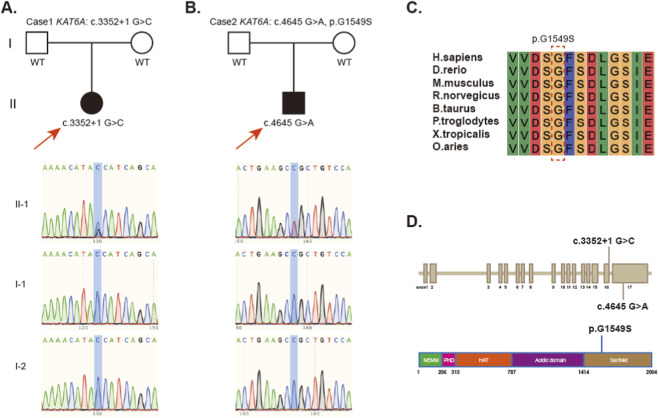
*De novo* variants in two cases with Arboleda-Tham syndrome. **(A)** Family verification of patient with *KAT6A* variant in case 1. **(B)** Family validation of patient with case 2 carrying *KAT6A* variant. **(C)** Conservation of the p. G1549 residue, highlighted in read dotted box, across various species (Source: Uniprot). **(D)** Schematic representation of *KAT6A* variants.

A *de novo* heterozygous variant, NM_ 006766.5: c.3352 + 1G>C, in the *KAT6A* gene, was identified in case 1. The results were further confirmed by Sanger sequencing (Figure 2A). This variant is located in the canonical +1 splice site and is predicted to disrupt normal splicing, leading to aberrant transcript processing and loss of function, which was classified as pathogenic (PVS1, PS2).

In case 2, we also found a *KAT6A de novo* heterozygous variant NM_ 006766.5: c.4645G>A (p.Gly1549Ser), which is highly conserved across species and results in the substitution of glycine with serine (Figures 2B,C). This missense variant is located in exon17 and the Ser/Met domain (Figure 2D). In silico prediction tools based on protein structure and evolutionary conservation yielded discordant pathogenicity predictions for this variant (MetaLR: 0.4365, REVEL: 0.412, and AlphaMissense: 0.9419). According to ACMG criteria, the variant was classified as likely pathogenic (PS2, PM2_P, PM1_P).

## 3 Discussion

ARTHS represents a prototypical syndromic intellectual disability disorder caused by pathogenic variants in the *KAT6A* gene. To date, 152 genetically confirmed postnatal cases (Bayat et al., 2025) and 3 prenatal cases (Yu et al., 2025) of KAT6A-related ARTHS have been reported in the literature. In postnatal cases, the youngest previously documented age of onset was 2 months. Our study describes two unrelated Chinese neonates with likely pathogenic or pathogenic *KAT6A* variants who exhibited severe clinical manifestations within the first month of life, thereby contributing to the growing recognition of early neonatal presentation of this syndrome and expanding its phenotypic and temporal spectrum.

Our cases demonstrate that cardiac arrhythmias and seizures can occur in neonates with *KAT6A* variants, establishing these as previously underrecognized early manifestations of this syndrome (Bae et al., 2021). The recognition of these complex features may facilitate earlier genetic diagnosis and identification, although the direct causative relationship between these manifestations and *KAT6A* variants requires further validation through larger cohort studies and mechanistic investigations. Regarding the cardiac arrhythmias observed in patient 1, it is important to note that supraventricular tachycardia with pre-excitation patterns occurs in 1/250–1/1,000 of infants with or without congenital heart disease (Maclennan et al., 2024), and is not uncommon in neonates with structural cardiac anomalies independent of genetic syndromes. Whether the arrhythmias in this case represent a direct consequence of KAT6A-mediated disruption of cardiac electrophysiological development or an expected complication of the underlying structural heart disease remains uncertain and requires further mechanistic investigation. All participating families provided comprehensive clinical data for publication but declined to share facial photographs due to medical privacy concerns.

Both patients exhibited profound developmental delays, with patient 1 demonstrating particularly notable language impairment characterized by markedly reduced verbal output at 3 years of age. The primary caregivers (grandparents) subjectively denied concerns about intellectual impairment that reporting only selective verbal reticence with unfamiliar individuals. However, these observations were objectively confirmed through standardized assessment using the Griffiths Mental Development Scale-Chinese, underscoring the importance of objective developmental evaluation over parental perception. This finding has significant implications for clinical practice, as early developmental intervention may optimize long-term outcomes in ARTHS. A standardized developmental assessment is scheduled for patient 2 during subsequent follow-up visits to evaluate intellectual development.

ARTHS demonstrates marked phenotypic heterogeneity, although the underlying mechanisms remain poorly understood. A comprehensive review of documented cases has identified a characteristic clinical profile, with neurodevelopmental manifestations present in the vast majority of patients (intellectual disability in 99% of cases, significant speech impairment in 78%, and hypotonia in 51%) (Bayat et al., 2025). The cardiac presentation observed in patient 1 expands the recognized phenotypic spectrum of KAT6A-related disorders. Although structural cardiac anomalies have been well-documented in 40%–50% of affected individuals (Tham et al., 2015; Bayat et al., 2025), the occurrence of arrhythmias during the neonatal period represents an infrequently documented manifestation of ARTHS. As discussed above, the pathophysiological relationship between *KAT6A* variants and neonatal arrhythmias remains incompletely characterized. While KAT6A’s role in cardiac conduction system development has not been extensively studied (Bayat et al., 2025), the relatively high background prevalence of SVT in neonates suggests that arrhythmias in patients with both *KAT6A* variants and structural heart disease may represent multifactorial etiology. Further mechanistic studies and larger cohorts are essential to clarify whether *KAT6A* dysfunction directly predisposes to arrhythmogenesis or whether observed arrhythmias primarily reflect complications of structural disease. These observations underscore the importance of implementing standardized cardiac evaluations incorporating both echocardiography and rhythm monitoring for all patients with *KAT6A* variants, regardless of structural findings. The neonatal seizures observed in patient 2 represent an early presentation of neurological manifestations in ARTHS. Although seizures have been reported in 20%–30% of affected individuals, they typically emerge during early childhood rather than in the neonatal period (Millan et al., 2016), and the association between neonatal-onset seizures and *KAT6A* variants remains to be further explored. Similarly, neonatal seizures can occur in the context of respiratory distress, metabolic derangements, or other comorbidities, which may or may not be directly attributable to *KAT6A* variants. While no metabolic or endocrine instability was identified in patient 2, and hemodynamic support was not required during seizure episodes, definitive causal inference would require larger case series and mechanistic studies. Zwaveling-Soonawala et al. (Zwaveling-Soonawala et al., 2017) documented neurological abnormalities in individuals with KAT6A syndrome, while early-onset seizures were not specifically emphasized.

Notably, patient 2 in our cohort, carrying the p. Gly1549Ser variant, exhibited clinical manifestations significantly earlier than two previously reported cases with the same variant. Specifically, one individual was diagnosis at 13 years of age (Kennedy et al., 2019) (male, with neonatal hypotonia, abnormal nose shape, cognitive/behavioral issues, poor or absent speech, high palate, small teeth) and the other was 17 years of age (Troisi et al., 2022) (female, with microcephaly, neonatal hypotonia, abnormal eyes/nose/mouth shape, cognitive impairment, poor or absent speech, gastrointestinal dysmotility/feeding difficulties). This observed phenotypic variability may potentially reflect the influence of genetic modifiers, environmental contributions, or variations in clinical assessment methodologies, underscoring the critical need for comprehensive genotype-phenotype correlation analyses in expanded patient cohorts.

The early diagnosis of ARTHS carries significant clinical and genetic counseling implications. During the prenatal period, a fetus carrying *KAT6A* variant may present with an interrupted inferior vena cava and fetal growth restriction (Yu et al., 2025). After birth, the identification of severe neonatal manifestations as potential presenting features of ARTHS should prompt clinicians to include *KAT6A* variants in the differential diagnosis of neonates presenting with developmental delays and dysmorphic features, particularly when accompanied by cardiac anomalies or neurological symptoms, recognizing that the syndrome may present earlier than previously appreciated. An early diagnosis enables the implementation of appropriate cardiac surveillance, optimization of seizures control, and initiation of developmental interventions during critical periods of neuroplasticity.

*KAT6A* functions as a histone acetyltransferase that regulates critical biological processes associated with tumorigenesis, including cancer cell proliferation and metastasis (Zemnou et al., 2025; Zheng et al., 2025). Substantial evidence has established its involvement in the pathogenesis of multiple human malignancies, including leukemia (Xu et al., 2022), breast cancer (Zhu et al., 2025), hepatocellular carcinoma (Baell et al., 2018), glioma (Lv et al., 2017), colorectal cancer (Xie et al., 2020) and ovarian cancer (Liu et al., 2021). The established oncogenic properties of somatic *KAT6A* alterations (Avvakumov and Côté, 2007) suggest potential cancer predisposition in individuals with germline variants, although this association requires systematic investigation. Consequently, long-term oncological surveillance should be considered for pediatric patients with germline *KAT6A* variants.

Our study also have some limitations. First, this study is limited by the small sample size. In fact, we reported only a single case of a patient with a *KAT6A* variant presenting with arrhythmias. Larger, preferably multicenter cohort studies are needed to clarify and validate the potential association between this clinical manifestation and *KAT6A* variants. Moreover, functional validation of the identified variants through *in silico* splicing prediction tools or experimental approaches was not conducted, which represents a limitation in confirming their direct pathogenicity.

In conclusion, our work documents the occurrence of neonatal arrhythmias and seizures in patients with *KAT6A* variants. These findings contribute to expanding the phenotypic and temporal spectrum of ARTHS by highlighting severe neonatal manifestations. Although *KAT6A* variants should be considered in the differential diagnosis of neonates presenting with complex multisystem presentations including cardiac and neurological symptoms accompanied by dysmorphic features, clinicians should be aware that a definitive causal relationship between specific neonatal manifestations (such as arrhythmias or seizures) and *KAT6A* variants remains unestablished and requires validation through larger-scale studies. The identification of this novel pathogenic variant expands the mutational spectrum of ARTHS and provides critical insights into the genotype-phenotype correlations underlying this clinically heterogeneous neurodevelopmental disorder.

## Data Availability

The data of our study are available on request from the corresponding author. The data are not publicly available due to privacy or ethical restrictions.
